# X-ray fluorescence microscopy exposure estimates using a single excitation energy

**DOI:** 10.1107/S1600577526004923

**Published:** 2026-06-16

**Authors:** Benjamin Roter, Andrew M. Crawford, Thomas V. O’Halloran, Chris Jacobsen

**Affiliations:** a Applied Physics Program Northwestern University Evanston IL60208 USA; b Department of Microbiology, Genetics and Immuology Michigan State University East Lansing MI48824 USA; c Department of Chemistry Michigan State University East Lansing MI48824 USA; d Elemental Health Institute Michigan State University East Lansing MI48824 USA; e Department of Physics and Astronomy Northwestern University Evanston IL60208 USA; f Chemistry of Life Processes Institute Northwestern University Evanston IL60208 USA; University College London, United Kingdom

**Keywords:** X-ray fluorescence, elemental detection, scanning fluorescence X-ray microscopy

## Abstract

Scanning fluorescence X-ray microscopy is widely used for quantitative mapping of elemental concentrations, including in studies of essential but low-concentration metals in cells, tissues, and organs. Practical studies often use a single incident photon energy to excite fluorescence from many elements. We present calculations of the number of incident photons per pixel required to detect a specified areal concentration of an element in the case of far-from-threshold excitation, along with the calculated radiation dose consequently imparted in a simple model tissue. We also show how certain approximations can lead to less accurate estimates. These calculations are not specific to one particular experiment, though we also provide a comparison with one experimental result. These results can be used to guide experimental planning for studies of the role of low-concentration elements in biological tissues.

## Abstract 2

We present calculations on the number of illuminating photons per pixel required for elemental detection using X-ray fluorescence, even with far-from-threshold excitation.

## 1. Introduction

X-ray fluorescence (XRF) allows for the identification of specific chemical elements, as was understood more than a century ago (Barkla, 1911 ▸; Moseley, 1913 ▸; Moseley, 1914 ▸). Scanning a small X-ray beam allows for the imaging of elemental content (Horowitz & Howell, 1972 ▸; Sparks, 1980 ▸; Jones *et al.*, 1984 ▸) in an approach which we refer to here as scanning fluorescence X-ray microscopy (SFXM). This approach shows wide utility including in research of the roles of essential but low-concentration metals in biological functions in 2D (Paunesku *et al.*, 2006 ▸; Fahrni, 2007 ▸; Pushie *et al.*, 2014 ▸), and in 3D via X-ray fluorescence tomography (de Jonge & Vogt, 2010 ▸).

In transmission X-ray microscopy using absorption and phase contrast, there is rich literature on calculations of X-ray photon fluence requirements for achieving a specified spatial resolution (Sayre *et al.*, 1977 ▸; Rudolph *et al.*, 1990 ▸; Schneider, 1998 ▸; Howells *et al.*, 2009 ▸; Du & Jacobsen, 2018 ▸), and similar calculations exist for imaging based on coherent scattering (Shen *et al.*, 2004 ▸; Schropp & Schroer, 2010 ▸). (While photometry defines fluence in terms of energy per area, these other studies use photon fluence or photons per area for calculations in which Poisson statistics of photon counts are important.) However, while there have been careful studies of the achieved elemental detection limits in specific synchrotron-based SFXM measurements (De Samber *et al.*, 2016 ▸; Adams *et al.*, 2011 ▸), there are no recent calculations predicting the off-threshold illumination required to achieve a specified level of detection. Several early studies used approximate values for the relevant interaction coefficients to compare X-ray-induced X-ray fluorescence against other elemental detection methods such as electron- or proton-induced X-ray emission, and electron energy-loss spectroscopy (Kirz *et al.*, 1978 ▸; Kirz, 1980*a* ▸). This methodology was also used to predict X-ray illumination requirements for SFXM (Kirz, 1980*b* ▸). More recent work considered radiation dose limits for imaging hydrated cells (Fayard *et al.*, 2009 ▸). However, these studies assumed illumination at a photon energy just above the relevant absorption edge of each element, which is not the practice of most studies today.

Today, SFXM is often carried out using a single incident photon energy (often 10–12 keV in the case of many biological studies) to excite the emission of X-ray fluorescence from multiple elements simultaneously, even though these elements have absorption edges and emission lines at photon energies well below the illumination photon energy. Photon counting at energies characteristic of the elements of interest, coupled with spectrum analysis, background correction, and mass calibration standardization, leads to quantitative elemental maps of the sample. For biologists interrogating tissue and cell-based samples, the pixel-by pixel quantitative resolution of heterogeneity in these elemental maps provides powerful insights into fundamental biological processes, as well as etiology of disease states (Zee *et al.*, 2022 ▸). To model this common practice and better understand limitations in the quantitative results, one must account for far-from-threshold excitation.

In most SFXM experiments, energy-dispersive spectrometry (EDS) detectors are used in conjunction with analysis programs (Vogt, 2003 ▸; Solé *et al.*, 2007 ▸; Ryan *et al.*, 2010 ▸; Crawford *et al.*, 2019 ▸) that account for the energy resolution of such detectors, and backgrounds including X-ray scattering and incomplete charge collection from the detector (Van Grieken & Markowicz, 2002 ▸). Fluorescence spectrum analysis is simplified if one instead uses wavelength dispersive spectrometry (WDS) detectors (De Pauw *et al.*, 2024 ▸), but WDS is limited in solid angle coverage and wavelength range so that it is usually not employed unless chemical state information is required.

With these developments, we revisit the question of illumination requirements for SFXM imaging of elemental concentrations. We account for illumination photon energies that can be far from thresholds for specific element excitation, and we make use of easy computer access to accurate tabulations of the relevant X-ray interaction coefficients as is described in Section 2. This allows us to predict the incident photon fluence 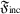 (given here in photons cm^−2^; see Table 1 ▸) at a single incident photon energy *E*_inc_ required to detect specific elements at a specific areal mass concentration ρ′. We note that the mass per area ρ′ is distinct from the density ρ of mass per volume; this follows the notation used in Section 9.2 of a recent text (Jacobsen, 2020 ▸) (see Section 3.4 of this manuscript for conversions to other metrics). Such calculations are especially interesting when considering a specified minimal value 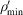, which sets the limit of detection (LOD) for an element. We can then use this photon fluence 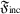 to calculate the radiation skin dose *D*_skin_ necessarily imparted to the incident-beam-facing surface of a specified matrix material (for example, for detection of an element located in a biological cell with some average composition), since dose can set limits on the imaging of radiation-sensitive materials. We show *xraylib*-based (Schoonjans *et al.*, 2011 ▸) calculations for a wide range of trace elements at different incident energies using an X-ray fluorescence forward model that considers the excitation dependence of mass photoionization cross sections, Coster–Kronig transitions, cascade effects, the presence of an EDS detector entrance window, and the presence of any gas in the sample environment.

These results can be used for experimental planning. If one knows the absolute incident photon energy *E*_inc_, incident flux (in photons s^−1^), focal spot size, and solid angle of detection of an energy-dispersive detector, one can calculate the per-pixel exposure time required to reach an incident number of photons per pixel 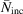 as needed to obtain a certain detectable mass per area ρ′ for a specified element.

## 2. The X-ray fluorescence forward model

In a typical SFXM experiment, the specimen is meant to be illuminated with 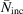 incident photons per pixel at energy *E*_inc_ (per-pixel statistical fluctuations will be distributed around 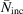). Some fraction of the incident photons are absorbed by a target element of atomic number *Z*, leading ultimately to a mean number of detected photons of 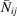 corresponding to X-ray fluorescence line *ij*, where *i* and *j* are initial and the final electron vacancy states, respectively. (For notational simplicity, we do not explicitly indicate the energy dependence of each variable, but this is shown in Table 1 ▸; see also Section S1 in the supporting information for information on relating transitions *ij* to conventional X-ray nomenclature.) For a specimen sufficiently thin that there is neither scattering of the incident signal nor self-absorption of the fluorescence signal, the mean number 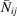 of detected fluorescence photons per pixel can be expressed as (Sherman, 1955 ▸; Schoonjans *et al.*, 2011 ▸; Kirz *et al.*, 1978 ▸; Sparks, 1980 ▸) 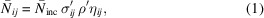
where 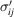 is the mass X-ray fluorescence production cross section (*e.g.* cm^2^ g^−1^) at energy *E*_inc_, ρ′ is the local areal mass density (*e.g.* g cm^−2^), and η_*ij*_ is the net detection efficiency for line *ij* [equation (5)].

In this work, we use mass cross sections 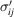 (typically given in cm^2^ g^−1^), which are distinct from atomic cross sections σ_*ij*_ (typically given in barns per atom, where 1 barn = 10^−24^ cm^2^). Mass cross sections are more common to find in fundamental parameter databases (Thompson *et al.*, 2009 ▸), but they are related to atomic cross sections via 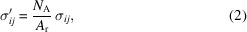
where σ_*ij*_ is the atomic XRF production cross section, *N*_A_ is Avogadro’s number, and *A*_r_ is the relative atomic mass (molar mass) of the absorbing element. Mass XRF production cross sections are related to the probability of fluorescence line *ij* being emitted due to subshell *i* being excited by an incident X-ray photon; this can be calculated via (Schoonjans *et al.*, 2011 ▸) 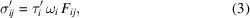
where 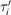 is the mass photoionization partial cross section of subshell *i*, ω_*i*_ is the subshell fluorescence yield, and *F*_*ij*_ is the fractional yield or branching ratio which is the fraction of ω_*i*_ emitted fluorescence photons corresponding to the *ij* line. For 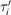, care is generally taken when determining what values to use. The simplest assumption is that, as one crosses the threshold energy for removing an electron from a specific subshell, the fractional increase in absorption tells one the fractional increase in fluorescence events resulting from that subshell. This gives rise to the jump ratio approximation *r*_*i*_ for subshell *i* (Martin, 1927 ▸; Compton & Allison, 1935 ▸; Sherman, 1955 ▸). This approximation generally holds well for *K* shell excitations, with the fraction of absorption events that go towards creating *K* shell vacancies given by 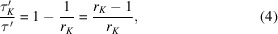
where τ′ is the total mass photoionization cross section. For the *L* shell and beyond, the excitation-dependent nature of photoionization becomes more important at higher *E*_inc_ relative to the respective subshell absorption edge (Scofield, 1973 ▸; Hönicke *et al.*, 2014 ▸; Hönicke *et al.*, 2016 ▸; Hönicke, 2023 ▸). Therefore, the jump ratio approximation of equation (4) loses accuracy when applied to *L* shell fluorescence, as illustrated in Section 4. Two additional phenomena (Bambynek *et al.*, 1972 ▸; Schoonjans *et al.*, 2011 ▸) play important roles for those shells:

(i) Coster–Kronig (CK) transitions: special cases of Auger electron emission where electron vacancies are filled by electrons in higher subshells within the same shell, causing electrons to be emitted from either higher shells or from the same shell. In the latter case, the CK transition becomes a super Coster–Kronig (SCK) transition.

(ii) Cascade effects: vacancies created due to general Auger emission, electrons emitted due to (S)CK transitions, and/or XRF events involving lower shells.

Together, these phenomena can affect the values of 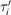, especially as *E*_inc_ goes well beyond edge energies 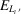 [where *i*′ = 1, 2, 3 (similar considerations apply to *M* edges and beyond)]; this is illustrated in calculations shown in Section 3.1.

Detection efficiencies η for X-ray fluorescence typically consider the solid angle fraction Ω/(4π) that a fluorescence detector subtends. Most hard X-ray fluorescence experiments use EDS detectors, and in most cases the cooled detection elements are protected from contamination by being placed behind thin windows. These windows of thickness *t*_w_ are often fabricated of beryllium so as to minimize the absorption of fluorescence at energies of a few keV or above. In addition to detector entrance windows, we also account for signal absorption in a gas path (air, helium, *etc*.) over a distance *d*_g_ between the sample and detector window. Therefore, we modify the efficiency η to include window and gas attenuation factors, giving η_*ij*_ for a particular fluorescence line *ij* of 
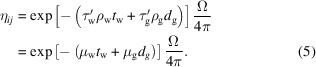
In the above equation, 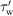 and 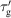 are the total mass photoionization cross sections of the window material and gas, respectively, *E*_*ij*_ is the fluorescence energy of line *ij* of an emitting element, and ρ_w_ and ρ_g_ are the densities of the window material and gas, respectively. [The second form of equation (5) uses the energy-dependent window and gas material linear absorption coefficients μ_w_ = 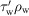 and μ_g_ = 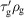, respectively.] We show in Section S3 of the supporting information how a beryllium window and air can change the minimum number of incident photons 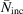 per pixel required to detect a given number of fluorescence photons per pixel at a specified areal mass concentration 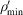. Incomplete charge collection (Van Grieken & Markowicz, 2002 ▸) of electron–hole separation events in the detector can also effectively reduce the number of detected photons; however, to simplify calculations, we ignore this factor.

## 3. Calculations of minimum photon exposure and radiation dose

We now use equation (1) to solve for the required number of incident photons 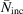 per area. We do so based on a requirement to detect 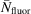 fluorescence photons per pixel in an image.

### 3.1. Minimum number of incident photons per pixel

To obtain the theoretical minimum number of incident photons per pixel 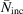 for detecting 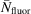 fluorescent photons per pixel, all fluorescence line contributions calculated using equation (1) can be summed up via 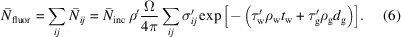
From basic considerations of false-positive and false-negative error rates in detection (Currie, 1968 ▸), it is often sufficient to detect 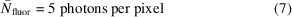
in order to detect the presence of an element at low concentration when the background is sufficiently low. The choice of 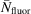 = 5 photons per pixel is somewhat arbitrary, though it is consistent with the Rose criterion (Rose, 1946 ▸) for image recognition in the case of zero background signal. The assumption of near-zero background indeed applies to the case of detecting low-concentration essential metals in biological specimens when using full-spectrum fitting methods to remove background signals; in our case we used the program *M-BLANK* (Crawford *et al.*, 2019 ▸) to obtain experimental histograms of detected photons with near-zero background signal as shown in Fig. 2 and discussed in Section 3.5.

With a specified requirement for 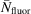, one can rearrange equation (6) to solve for the required number of incident photons per pixel 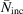. We do so at a specified minimum value of detectable mass density 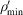 corresponding to an LOD; this gives 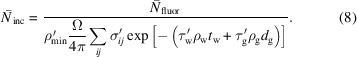
This result allows us to predict the number of photons 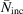 at photon energy *E*_inc_ required per pixel when attempting to detect a mass concentration 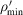 of any specified element.

### 3.2. Corresponding radiation dose to a matrix material

In many studies, one is measuring a low mass concentration 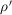 of a specified element present in a higher-concentration matrix material; one example involves study of the role of zinc in oocytes and embryos during fertilization (Que *et al.*, 2015 ▸; Kong *et al.*, 2015 ▸; Balough *et al.*, 2025 ▸). High radiation doses can lead to morphological changes and mass loss in the organic materials in cells and tissues (Jacobsen, 2020 ▸), so it is also important to provide an estimate of the radiation dose imparted to a matrix material (which we denote with the subscript ‘mat’) associated with irradiation with 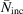 photons per area. To do so, we first consider the incident photon fluence 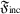 of 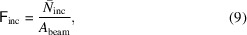
where *A*_beam_ is the area of the incident beam [which equals π(*d*_beam_/2)^2^ for a uniform circular beam spot of diameter *d*_beam_]. Inserting the result of equation (8) into this expression yields 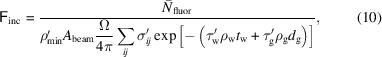
where 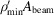 is also known as the minimum detectable mass 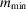 (Kirz *et al.*, 1978 ▸). Skin dose *D*_skin_ is the radiation dose delivered to the beam-facing surface of a matrix material; it can be found from (Kirz *et al.*, 1978 ▸; Jacobsen, 2020 ▸) 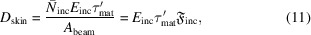
where (Jacobsen, 2020 ▸) 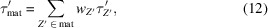
and where the final form of equation (11) comes from equation (9). In the above two equations, 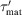 is the total mass photoionization cross section of the sample matrix, and 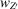 is the weighting coefficient accounting for the atom number fraction of each element *Z*′ present in the matrix material (that is, for each *Z*′ ∈ mat). If the sample is tilted by angle θ relative to the transverse of the incident beam (so as to balance between XRF self-absorption minimization and beam broadening), then the beam width as seen by the tilted sample pixels along one direction increases by a factor of 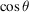. In this case, equation (11) becomes 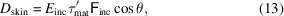
leading to a factor 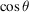 drop in the skin dose. Substituting equation (10) into the above equation results in 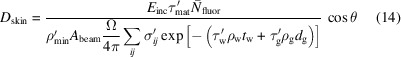
as the skin dose.

It is common in X-ray imaging calculations to represent biological specimens as comprising a model protein with the compositional average of all 20 amino acids, with a stoichiometric composition of H_48.6_C_32.9_N_8.9_O_8.9_S_0.6_ (London *et al.*, 1989 ▸) (the density does not need to be specified; see Section S2 in the supporting information). We used that protein as the matrix material in the skin dose calculations described in Section 3.3.

### 3.3. Numerical example: low-concentration elements in a protein matrix

In Section 3.1, we derived the minimum number 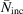 of incident X-ray photons per pixel [equation (8)] required for detection of an elemental concentration 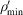. As noted in Section 3.1, those derivations were performed under the assumption that the detection of 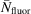 = 5 photons per pixel is sufficient for elemental detection (this point is discussed further in Section 3.5). Because this covers most SFXM studies today, our calculations only include X-ray fluorescence from *K* and *L* subshells; our approach could be extended to *M* subshells and beyond if desired. From the calculations of 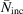, we also computed the corresponding matrix material skin dose *D*_skin_ [equation (14)] as described in Section 3.2. We used those results to obtain numerical estimates representative of typical experiments while assuming the following:

(i) The specimen matrix is the model protein of stoichiometric composition H_48.6_C_32.9_N_8.9_O_8.9_S_0.6_ as discussed in Section 3.2.

(ii) The specimen is illuminated with a single incident photon energy *E*_inc_.

(iii) The specimen contains trace elements *Z* ∈ [10, 92], all at an areal mass concentration of 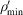 = 0.05 µg cm^−2^. This value of 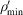 is representative of the limit of detection in an SFXM experiment. Conversions to other metrics for elemental sensitivity are given in Section 3.4.

(iv) The specimen is housed in a vacuum environment so that *d*_g_ = 0.

(v) For each element *Z*, a fluorescence signal with 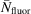 = 5 photons per pixel must be counted by a windowless detector with an acceptance solid angle of Ω = 1.35 steradians (sr).

These assumptions were sufficient to calculate the required number of incident photons 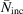 per pixel. For computing the resulting skin dose *D*_skin_ in the matrix material, we used a value of *A*_beam_ corresponding to a circular beam focus *d*_beam_ = 40 nm in diameter, and we assumed that the specimen was at normal incidence to the beam so that θ = 0.

Our calculations utilized tabulations provided by the *xraylib* fundamental parameter database (Schoonjans *et al.*, 2011 ▸). That database contains information relevant for *K*-, *L*-, and *M*-shell fluorescence and involves a complete XRF forward model that accounts for the excitation dependence of mass photoionization partial cross sections (PCSs), CK transitions, and cascade effects. The *xraylib* database does not include some weak fluorescence lines, like *K*α_3_ and *L*β_2_, that are formally forbidden by selection rules in single electron theory (Dyson, 1973 ▸) (though they can in fact be weakly present). The database also does not include non-radiative transitions such as SCK transitions. These omitted parameters would not lead to noticeable changes in our results if they were somehow to be included.

**Figure 1:**
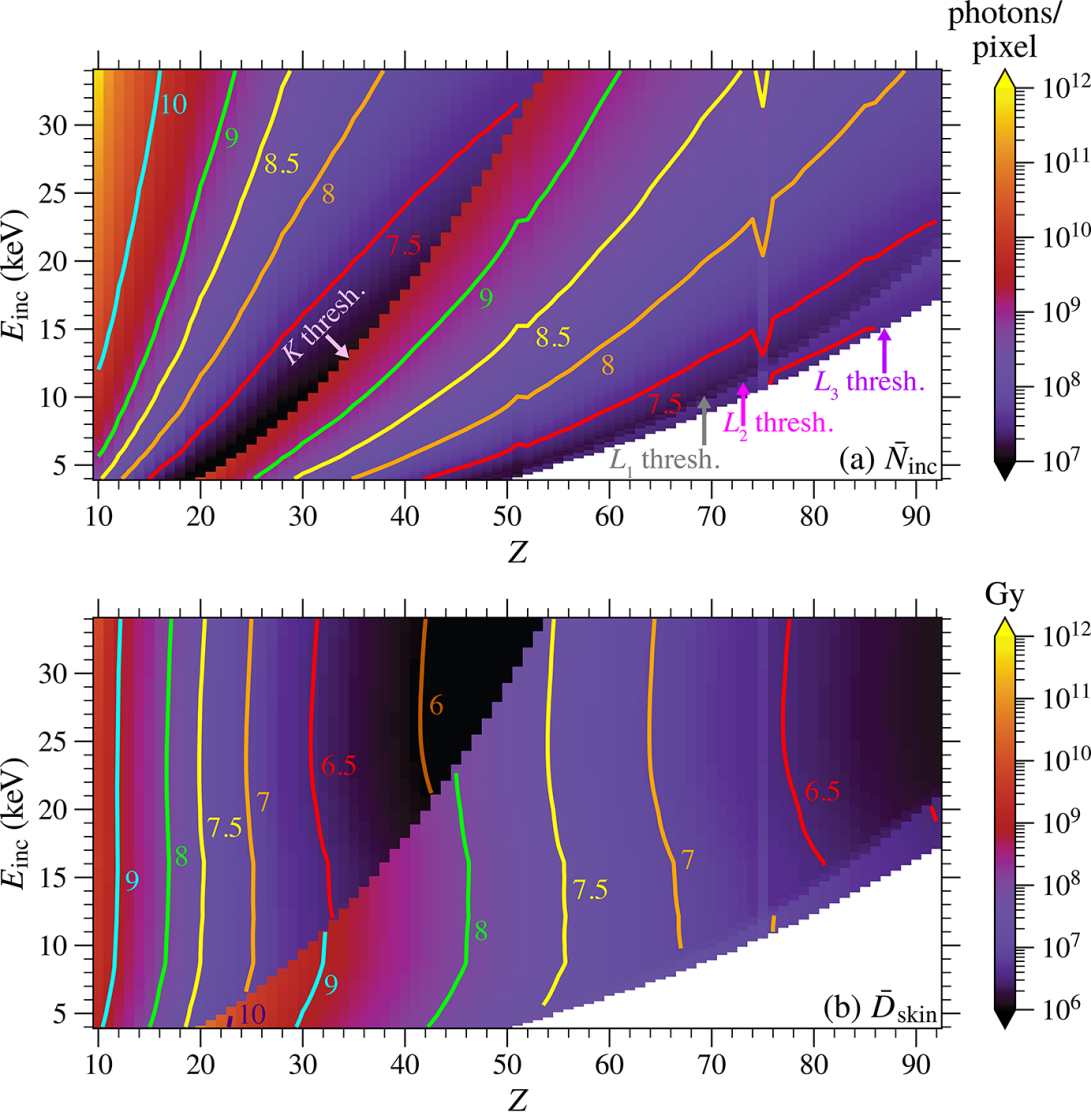
Combined false color maps and contour plots of the expected minimum number of incident photons 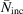 per pixel (*a*) and skin dose *D*_skin_ in Gray imparted (*b*) for element detection in vacuum. These values are shown versus trace element atomic number *Z* and individual incident photon energies *E*_inc_. These calculations were carried out for a limit of detection of 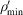 = 0.05 µg cm^−2^ and for the detection of 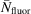 = 5 X-ray photons per pixel summed over all accessible *K* and *L* fluorescence emission lines. We assumed that the X-ray fluorescence detector was windowless and had a solid angle of collection of Ω = 1.35 sr. The skin dose *D*_skin_ (*b*) associated with 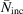 was calculated assuming a model protein composition of H_48.6_C_32.9_N_8.9_O_8.9_S_0.6_ (London *et al.*, 1989 ▸) and a focused beam diameter of *d*_beam_ = 40 nm. These calculations included all the effects related to photoionization partial cross sections as described in Section 3.1. As *Z* increased, the contributions to 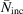 and *D*_skin_ for a particular subshell abruptly changed when *E*_inc_ hit and exceeded that subshell’s absorption edge (labeled ‘thresh.’). The white regions in each panel exist due to an individual value of *E*_inc_ not being high enough to excite the *L*_3_ subshell, as well as due to the exclusion of XRF events stemming from shells greater than *L*_3_ from our calculations. This calculation employed tabulated data from *xraylib* (Schoonjans *et al.*, 2011 ▸). Contour values correspond to base-10 exponents.

With the above assumptions and fundamental parameter tabulations in hand, we show in Fig. 1 ▸ our calculated values of 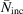 (*a*) and *D*_skin_ (*b*) as a function of atomic number *Z*, as well as a function of individual incident photon energies *E*_inc_ over the range 4 to 34 keV. The values of 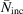 and *D*_skin_ are shown using a false color map, with the color map scale shown on the right. One can think of this as a topographical map of terrain, with contour lines at altitude intervals. The contour lines are labeled with numbers *C*, which correspond to values of 10^*C*^ for 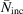 and *D*_skin_. The calculations utilized *K* and *L* fluorescence lines only, so the white region at lower right reflected incident photon energies *E*_inc_ that were too low to reach the threshold for exciting *L* line fluorescence. In a similar fashion, the plots showed a ‘topographical cliff’, or sharp decrease, in both 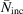 (*a*) and *D*_skin_ (*b*) when *E*_inc_ increased to reach the threshold for exciting *K* fluorescence; this cliff started at (*Z* = 20, *E*_inc_ = 4 keV) and rose to (*Z* = 54, *E*_inc_ = 34 keV).

Limits on radiation dose to the specimen depend very much on specimen preparation conditions. For room-temperature hydrated specimens, radiation doses as low as 10^5^ Gy can lead to mass loss of organic components and shrinkage, even in chemically fixed specimens (Williams *et al.*, 1993 ▸). Frozen hydrated samples imaged at liquid nitrogen temperature are far more robust, with little change seen between successive 20 nm resolution images at radiation doses of about 3 × 10^7^ Gy (Deng *et al.*, 2017 ▸). Even at these doses, the damage is mainly in the form of bond breaking in organic materials, with minimal mass loss (Beetz & Jacobsen, 2003 ▸). For studies of elemental distributions in biological samples, samples that have been rapidly frozen (often via plunge-freezing in liquid ethane or propane) and then freeze-dried show excellent retention of low-concentration metals (Perrin *et al.*, 2015 ▸; Jin *et al.*, 2017 ▸). Samples in such a condition show little mass loss at doses probably up to about 10^9^ Gy, though there is a lack of systematic studies exploring this. Since Fig. 1 ▸ showed that radiation doses above 10^8^ Gy are rarely required, one can expect that radiation dose will not usually impede X-ray fluorescence microscopy studies of elemental distributions.

While radiation damage depends mainly on total dose rather than dose rate, sample heating depends mainly on both dose rate and heat conduction in the specimen and to the specimen mount. Sample heating has not often been observed in X-ray microscopy of biological specimens, but finite element analysis calculations have suggested that nanofocused hard X-ray beams can lead to localized specimen heating at incident photon fluxes above about 10^11^ photons s^−1^ (Wallander & Wallentin, 2017 ▸).

### 3.4. Other equivalent measures of elemental detection

We have carried out our calculations based on areal mass density ρ′ since that is the quantity that is directly useful for the forward model described in Section 2. However, many users of SFXM prefer to use other metrics for elemental detection. As noted in Section 3.2, the minimum detectable mass 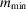 is 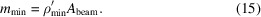
If the beam intensity is uniform in a circle of diameter *d*_beam_, we have 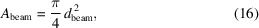
which gives 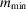 = 0.628 ag (*i.e.* 6.28 × 10^−19^ g) for 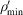 = 0.05 µg cm^−2^ and *d*_beam_ = 40 nm. The minimum number of detectable atoms 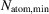 for a fluorescing element with molar mass *A*_r_ is then 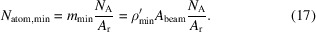
For 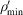 = 0.05 µg cm^−2^, *d*_beam_ = 40 nm, and a molar mass of *A*_r_ = 40.08 g mol^−1^ for Ca, this gives a minimum number of detected atoms of 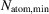 = 9440 atoms. If this number of atoms is in a matrix material of density ρ_mat_, thickness *t*_mat_, and molar mass *A*_r,mat_, the number of matrix atoms *N*_atom,mat_ is given by 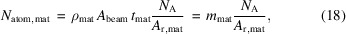
where *m*_mat_ is the absolute mass of the matrix. A matrix of carbon with ρ_mat_ = 2.26 g cm^−3^, *A*_r,mat_ = 12.011 g mol^−1^, and thickness *t*_mat_ = 10 µm thus has *N*_mat_ = 1.4 × 10^9^ atoms in the illuminated region. From equations (17) and (18), one can calculate the atomic parts-per-million sensitivity 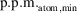 as 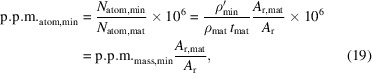
where 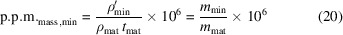
is the traditional mass parts-per-million sensitivity. For this example here of calcium in a carbon matrix, these two equations yield 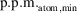 = 6.63 p.p.m. and 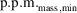 = 22.1 p.p.m., respectively.

### 3.5. Experimental validation

The calculations of the minimum number of incident photons per pixel 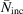 of equation (8) assumed zero background when detecting 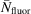 fluorescence photons per pixel. In a typical SFXM experiment, scattering in the forms of elastic (Rayleigh) and inelastic (Compton) scattering can lead to spectral background peaks at and slightly below incident energy *E*_inc_, respectively, and incomplete charge collection in energy-dispersive detectors can also appear as a background signal (Van Grieken & Markowicz, 2002 ▸). However, as mentioned in Section 1, full-spectrum analysis programs are capable of correcting for these backgrounds (Ryan, 2000 ▸; Vogt, 2003 ▸; Solé *et al.*, 2007 ▸; Crawford *et al.*, 2019 ▸). While there are many reports of minimum detection limits in SFXM (Adams *et al.*, 2011 ▸; De Samber *et al.*, 2016 ▸), they usually are not accompanied by absolute measurements of photon fluence as required to compare experiments with these calculations for number of incident photons 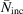 per pixel and a minimum detected areal mass concentration 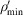.

We compare here with one recent experiment (Roter *et al.*, 2026 ▸) of *K* fluorescence of five low-concentration elements present in an SFXM experiment. This experiment involved a 10 µm-thick section of dehydrated mouse kidney tissue mounted on a Si_3_N_4_ window, imaged at beamline 8-BM-B at the Advanced Photon Source at Argonne National Laboratory, USA. In a typical scan with a per-pixel imaging time of *t*_dwell_ = 50 ms, the sample was illuminated with 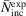 = 3.9 × 10^8^ photons per pixel (±5%) at *E*_inc_ = 10 keV photon energy. The full fluorescence spectrum was obtained using a seven-element energy-dispersive detector with an entrance window of *t*_w_ = 25 µm-thick beryllium and with a vacuum gap of *d*_v_ = 0.4 cm between the central detector sensor and the beryllium window. Unfortunately, the sample-to-detector plane distance 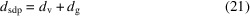
(where *d*_g_ is the air gap between the sample and beryllium window) was not directly measured due to the presence of a collimator guarding against stray X-ray scattering; instead, an inverse-square law fit of the signal at three different detector distances (Roter *et al.*, 2026 ▸) was used to obtain an estimate of the sample-to-sensor distance. This gave an estimate of *d*_sdp_ = 0.98 cm for the center detector sensor element, corresponding to an acceptance solid angle of Ω = 1.35 sr [see Section S3 of the supporting information of Roter *et al.* (2026 ▸); this estimate is discussed below]. The values of Ω and *E*_inc_ here correspond to the calculation assumptions described in Section 3.3. The recorded spectrum was analyzed with the *M-BLANK* software package (Crawford *et al.*, 2019 ▸) using spectral data obtained from a sample-free Si_3_N_4_ window for background subtraction, as well as elemental areal mass concentrations obtained by comparison with fluorescence signals obtained from an AXO 10X thin film standard (RF8-200-S2454, Applied X-ray Optics, GmbH). In that experiment, P, S, Ca, Fe, and Ni were all present in a wide range of concentrations. The forward model of equation (1) assumed no background other than from Poisson fluctuations stemming from the detection of fluorescence photons themselves; this well approximated the case of the selected elements since their true XRF signals were either originally much stronger than that of the experimental background or enough of the background was subtracted out when initially fitting raw fluorescence spectra.

For the five selected elements, we defined the limit of detection 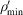 via background-corrected fits to the fluorescence of the Si_3_N_4_ window. After measuring the fluorescence emitted from an empty Si_3_N_4_ window, we averaged the resulting spectrum over all pixels to acquire a representative background spectrum. This average background was then subtracted at every pixel of the kidney section scan, and we fit the acquired difference spectra using the same *M-BLANK* parameterized peak model employed for the sample data (Crawford, 2020 ▸). This yielded a population of fitted, background-corrected signals (expressed as calibrated areal mass concentrations ρ′) across all substrate pixels for each element. We took the standard deviations 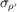 of those distributions as the noise levels, and defined the LOD to be 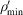 = 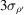. This approach provided an element-specific, data-driven estimate of 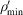 under the same experimental conditions and fitting model as the sample measurements.

To obtain measures of the total number of fluorescence photons *N*_fluor_ collected at each pixel within 1% of the per-element 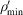 determined above, we defined energy windows for summing *K*α and *K*β photons using *xraylib* tabulated line energies *E*_*ij*_ (Schoonjans *et al.*, 2011 ▸) combined with an empirically calibrated detector response function. We specified the nominal photon energies of the *K*α_1_, *K*α_2_, *K*β_1_, and (where relevant) *K*β_2_ lines and treated those values as line centroids. Afterward, for each detector element, we parameterized the line energy resolution Δ*E*_*ij*_ as an energy-dependent full width at half-maximum (FWHM) via a standard Fano-limited model of (Schlosser *et al.*, 2010 ▸) 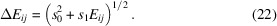
In the above equation, *s*_0_ and *s*_1_ are detector element-specific parameters obtained by fitting the measured detector element response to multiple fluorescence lines in the same dataset. We then defined the integration window bounds 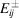 for each fluorescence line *ij* according to 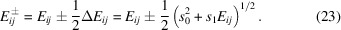
Because all values of *s*_0_ and *s*_1_ were extracted directly from Si_3_N_4_ scans rather than from a fixed lookup table, the resulting energy windows accurately reflected the actual detector performance under the specific beamline and low-count-rate conditions used in the experiment. For calcium, where the *K*α peaks have some overlap with potassium *K*β lines when the peak broadening of the energy-dispersive detector is taken into account, we mitigated interference by excluding pixels in which the fitted potassium concentration map exceeded its own value of 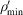 scaled up by the expected XRF intensity ratio *F*_*K*α_/*F*_*K*β_ = 8.65 (Schoonjans *et al.*, 2011 ▸). This ensured that the calcium photon statistics near the calcium limit of detection were not dominated by potassium *K*β spill-over (Crawford *et al.*, 2018 ▸).

**Figure 2:**
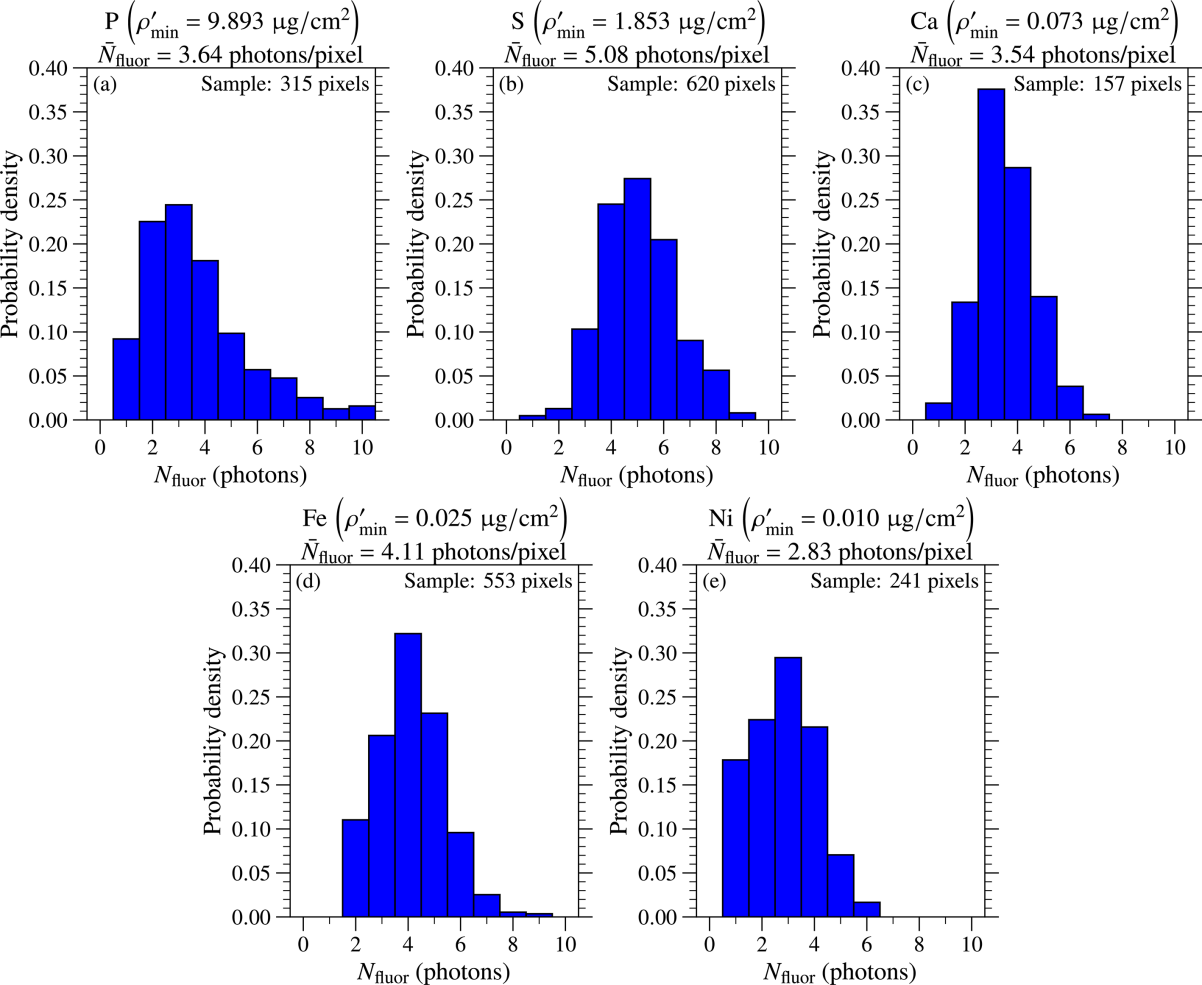
The distribution of total X-ray fluorescence photons detected in each pixel around different elemental LODs 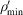 in our experiment at beamline 8-BM-B (Roter *et al.*, 2026 ▸). Shown here are histograms of probability densities for several elements with respect to the total number *N*_fluor_ of XRF photons detected for sample pixels within 1% of each element’s LOD, which we obtained by summing up the contributions from all detector elements. From those distributions, we were able to calculate the mean minimum number of X-ray fluorescence photons 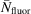 detected per pixel.

After calculating all energy windows, we summed up the total number of photons collected over all detector elements for each pixel within those windows to obtain an aggregate number *N*_fluor_ of XRF photons collected over all detector elements for each pixel around 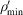. Ultimately, all of this resulted in a distribution of collected fluorescence photons *N*_fluor_ across all selected pixels, shown as histograms in Fig. 2 ▸. From these histograms of probability densities for each element, we obtained the mean number of XRF photons 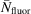 collected per pixel. In some cases, these mean values were slightly less than the somewhat arbitrary assumption of 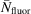 = 5 used as the basis for our calculations, highlighting the very low background that remains after using full-spectrum X-ray fluorescence analysis programs (Vogt, 2003 ▸; Solé *et al.*, 2007 ▸; Ryan *et al.*, 2010 ▸; Crawford *et al.*, 2019 ▸).

In Table 2 ▸, we show for the five selected elements both the limits of detection 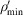 and the mean number of detected X-ray fluorescence photons 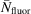 obtained from experimental data using the procedures described above. The table also shows corresponding values of 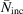 as calculated from equation (8) for those sample elements with two different distances *d*_g_ in air. We compared these results via the ratio 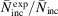 while using the previously reported value of 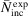 = 3.9 × 10^8^ photons per pixel (Roter *et al.*, 2026 ▸).

As noted earlier, we were unable to directly measure the sample-to-detector plane distance *d*_sdp_ of equation (21) for the central detector sensor element; therefore, we fit the signal to an inverse-square law at three different detector displacements Δ*d* and estimated that value to be *d*_sdp_ = 0.98 cm [see Section S3 of the supporting information of Roter *et al.* (2026 ▸)]. If the distance *d*_sdp_ were in fact to be a larger value, this would both increase the air gap distance *d*_g_ of equation (21) and decrease the detector’s solid angle of collection from the assumed value of Ω = 1.35 sr. Unlike the case of our theoretical calculations, which assumed a vacuum environment between the sample and detector (Section 3.3), the experimental result (Roter *et al.*, 2026 ▸) used an air environment, so one has greater absorption of fluorescence emission over the air gap distance *d*_g_ at lower photon energies relative to higher photon energies. It is possible that our three-distance inverse-square law fit for estimating *d*_sdp_ = 0.98 cm was erroneous. If instead we assume *d*_sdp_ = 4.5 cm, then the detector solid angle would drop to Ω = 0.13 sr, and the ratio 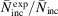 would be much closer to 1 for all detected fluorescence signals as discussed in Section S4 of the supporting information.

## 4. Effects of subshell excitation models

The calculation results shown in Table 2 ▸ incorporated the excitation dependence of subshell mass photoionization cross sections 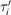, the existence of CK transitions, and the existence of both radiative and nonradiative cascade effects (see Sections 1 and 2). We now consider the changes that would arise with less-exact calculations. For these illustrations, we used a single incident photon energy of *E*_inc_ = 10 keV, and we assumed that 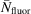 = 5 fluorescent photons per pixel were required to detect an areal concentration of 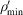 = 0.05 µg cm^−2^ when using a windowless EDS detector.

**Figure 3:**
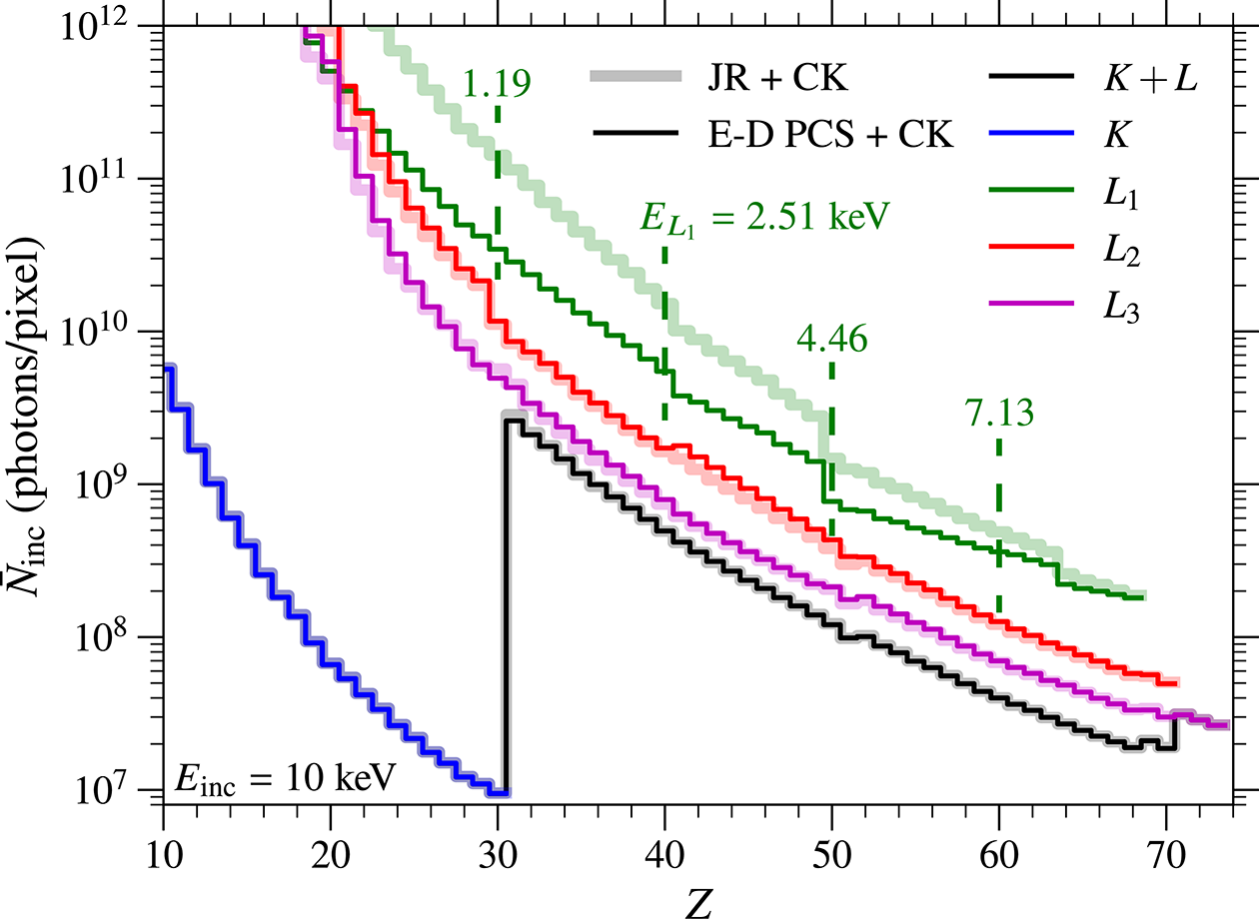
Subshell-specific calculations of the minimum number of incident photons 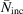 per pixel for a fixed incident photon energy of *E*_inc_ = 10 keV, with and without the ‘jump ratio’ approximation. The (E-D PCS + CK) calculations were carried out using excitation-dependent mass photoionization partial cross sections, while the less-accurate (JR + CK) calculations were carried out using the ‘jump ratio’ approximation of equation (4). In both cases, Coster–Kronig transitions were included (CK). As can be seen, the ‘jump ratio’ approximation leads to inaccurately high calculated values of 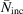, in particular when considering fluorescence from *L*_1_ lines when the excitation energy *E*_inc_ is well above the edge energy 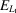. We assumed a windowless EDS detector and a vacuum environment for these calculations so as to not affect 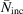 at lower *L* fluorescence emission energies. Tabulated data from *xraylib* (Schoonjans *et al.*, 2011 ▸).

To illustrate the shortcomings of using the simpler ‘jump ratio’ model of equation (4), we show in Fig. 3 ▸ differences in requirements for the minimum number of excitation photons 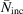 with and without this simpler model. As can be seen, the ‘jump ratio’ approximation leads to only small differences in 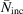 when detecting *K* fluorescence lines, but it leads to erroneously high estimates of 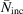 for the *L* shell. This was especially true when relying on the *L*_1_ line emission for elemental detection, as CK transitions cannot occur in that subshell. For that case, the error increased with greater differences between the incident photon energy *E*_inc_ and the absorption edge energy 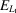 of a particular element. Differences like these have been experimentally observed (Hönicke *et al.*, 2014 ▸; Hönicke, 2023 ▸). In one example, inaccurate quantification of the thickness of a palladium (Pd) thin film was observed as the incident photon energy *E*_inc_ was increased well beyond the energy of each of the three *L* absorption edges; this demonstrated the inaccuracy of the jump ratio approach, in particular when using *L*_1_ fluorescence emission lines (Hönicke *et al.*, 2016 ▸).

**Figure 4:**
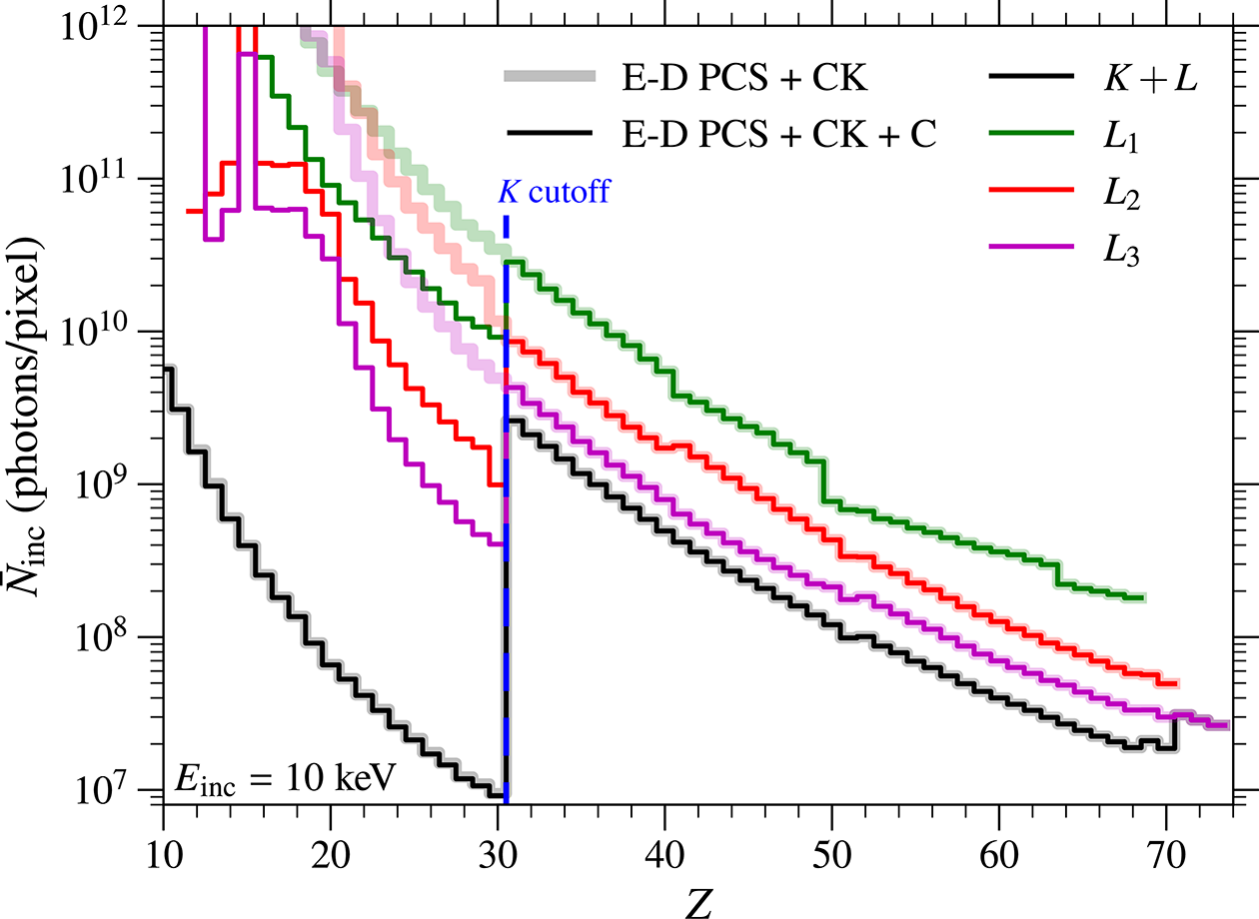
Subshell-specific calculations of the minimum number of incident photons 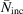 per pixel for a fixed incident photon energy of *E*_inc_ = 10 keV with and without the incorporation of cascade effects. Cascading due to *K* shell photoionization ceased when the *K* edge energy (labeled ‘*K* cutoff’) exceeded *E*_inc_, which occurs for *Z* ≥ 31 when *E*_inc_ = 10 keV. Double vacancies caused by electrons emitted during *L*-shell super Coster–Kronig transitions were omitted; thus, there were no cascade effects at all past the ‘*K* cutoff’ shown. Shown here are the results when cascade effects (both radiative and nonradiative) are both considered (E-D PCS + CK + C) and ignored (E-D PCS + CK). In both cases, we used energy-dependent partial cross sections (E-D PCS) and included Coster-Kronig transitions (CK). We assumed a windowless EDS detector and a vacuum environment for these calculations so as to not affect 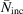 at lower *L* fluorescence emission energies. Tabulated data from *xraylib* (Schoonjans *et al.*, 2011 ▸).

The exclusion of cascade effects can also lead to erroneous estimates of the required number of incident photons 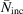 when considering *L* fluorescence emission lines, as shown in Fig. 4 ▸. For that comparison, 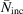 was lower for all values up until *Z* = 30, at which point *E*_inc_ = 10 keV is too low to excite the *K* edges of higher-*Z* elements. Because SCK transitions in the *L* shell were omitted, all values of 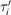 above *Z* = 30 were the same as their non-cascading counterparts. (Again, those values would not change significantly if they were included somehow.)

In Figs. 3 ▸ and 4 ▸, we observed differences in the estimates of 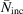 based only on considering *L* subshells. However, those differences effectively disappeared when considering the sum of all *K* and *L* shell fluorescence contributions.

## 5. Discussion

In our calculation results shown in Fig. 1 ▸ for detecting 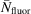 = 5 XRF photons per pixel at an LOD of 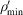 = 0.05 µg cm^−2^, the minimum number of incident photons 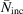 per pixel decreased with increasing atomic number *Z* for a given incident energy *E*_inc_, matching the expected trend: as *E*_inc_ reaches and exceeds an element’s absorption edge, the total mass photoionization cross section τ′ for that element increases, making photoionization (and therefore X-ray fluorescence) more probable. Correspondingly, the empirical XRF histograms of Fig. 2 ▸ showed that detecting roughly three to six fluorescence photons per pixel was consistently sufficient to reach the operational limits of detection across all elements examined. Therefore, our assumption of 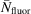 = 5 photons per pixel [equation (7)] was reasonable.

Comparison with experiment showed that our calculations are not disconnected from reality. While there are examples of experimental determination of achieved elemental sensitivity (Adams *et al.*, 2011 ▸; De Samber *et al.*, 2016 ▸), these examples do not provide sufficient information on absolute incident photon flux for a comparison like that shown in Table 2 ▸. The experimental results (Roter *et al.*, 2026 ▸) used for the comparison in Table 2 ▸ were also not perfect from the point of view of determining absolute incident flux, in that a photodiode measurement of incident flux was carried out as a separate measurement rather than during actual fluorescence sample scanning. In addition, we did not directly measure the distance between sample and detector entrance window *d*_g_ as discussed in Section 3.5; if we assume a revised value of *d*_sdp_ = 4.5 cm (leading to *d*_g_ = 4.1 cm) as discussed in Section S4 of the supporting information, Table 2 ▸ gives good agreement between experiment and calculations. This potential discrepancy, and its possible resolution, highlights the value of careful measurements of absolute incident flux, sample-to-detector distance, and fluorescence detector solid angle of signal collection in future work.

The calculated results of 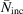 of equation (6) and *D*_skin_ of equation (13) are easy to adjust for parameter choices other than those used in Section 3.3 since both 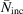 and *D*_skin_ have a linear dependence on most of the input parameters. Thus, one can easily adjust the numerical values shown in Fig. 1 ▸ in a linear way to account for different values of the required number of detected fluorescent photons 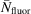 per pixel, the detector’s solid angle acceptance Ω, and the elemental limit of areal mass detection 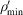. The material and thickness of any detector window or gas path in the experiment between specimen and detector appear in a nonlinear fashion in equations (6) and (13).

## 6. Conclusion

We have presented here an approach to estimate the minimum number of incident photons 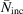 per pixel required for the detection of low-concentration elements when using a single incident photon energy *E*_inc_ to excite X-ray fluorescence from many different elements *Z*, which is representative of most experiments in SFXM. Earlier calculations (Kirz, 1980*b* ▸) assumed the use of an incident photon energy just above an element’s absorption edge, ideal for detecting just one element. In addition, we made use of *xraylib* (Schoonjans *et al.*, 2011 ▸), which provides computer-accessible tabulations of all relevant parameters and thus allows for more complete calculations. As a result, our model accounted for the incident energy dependence of mass photoionization partial cross sections, CK transitions, cascade effects, and attenuation due to detector windows and gas within a sample environment. As technology upgrades at synchrotrons lead to higher photon brightness, estimates of the limits of detection play an increasingly important role in the planning of SFXM experiments, laying the groundwork for next-generation discoveries in many areas including inorganic physiology.

## 7. Related literature

The following references, not cited in the main body of the paper, have been cited in the supporting information: Deslattes (1969 ▸); Henke *et al.* (1993 ▸); Jenkins *et al.* (1991 ▸); McCullough (1975 ▸); Siegbahn (1925 ▸).

## Supplementary Material

Sections S1 to S4 including Figures S1 and S2. DOI: 10.1107/S1600577526004923/ing5024sup1.pdf

## Figures and Tables

**Table 1:** Energy-dependent parameters used in our calculations, with descriptions, indications of where they first appear, and units provided

Term	Energy dependence	Description	Units
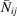	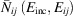	Mean number of detected fluorescence photons for a given fluorescence line [equation (1)]	photons per pixel
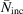	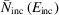	Mean number of incident photons illuminating a specimen [equation (1)]	photons per pixel
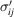	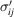 (*E*_inc_)	Mass X-ray fluorescence line production cross section [equation (1)]	cm^2^ g^−1^
η_*ij*_	η_*ij*_(*E*_*ij*_)	X-ray fluorescence line net detection efficiency [equations (1) and (5)]	Unitless
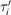	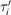 (*E*_inc_)	Mass photoionization partial cross section of subshell *i* [equation (3)]	cm^2^ g^−1^
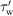	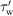 (*E*_*ij*_)	Total mass photoionization cross section of a detector window material [equation (5)]	cm^2^ g^−1^
μ_w_	μ_w_(*E*_*ij*_)	Linear absorption coefficient of a detector window material [equation (5)]	cm^−1^
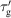	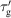 (*E*_*ij*_)	Total mass photoionization cross section of any gas in the specimen environment [equation (5)]	cm^2^ g^−1^
μ_g_	μ_g_(*E*_*ij*_)	Linear absorption coefficient of any gas in the specimen environment [equation (5)]	cm^−1^
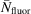	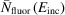	Total mean number of detected fluorescence photons [equation (6)]	photons per pixel
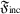	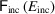	Photon fluence incident upon a specimen [equation (10)]	photons cm^−2^
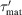	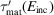	Total mass photoionization cross section of a matrix material [equation (12)]	cm^2^ g^−1^
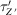	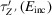	Total mass photoionization cross section of element *Z*′ in a matrix material [equation (12)]	cm^2^ g^−1^
*D*_skin_	*D*_skin_(*E*_inc_)	Radiation skin dose imparted to a matrix material’s beam-facing surface [equation (14)]	Gy

**Table 2:** Comparison of results from one particular experiment (Roter *et al.*, 2026 ▸) (with 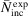 from Section 3.5) against calculated values for mass concentrations 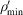 and detected number of fluorescent photons 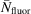 per pixel, along with the number of incident photons 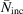 per pixel. As noted in Section 3.5, we did not have a direct measure of the air gap distance *d*_g_ of equation (21); therefore, we show the ratio of experimental to theoretical incident photons 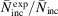 for the sample *d*_sdp_ = 0.98 cm from the detector plane, as well as for a revised value of *d*_sdp_ = 4.5 cm (see Section S4 of the supporting information)

Element	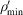 (µg cm^−2^)	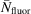 (photons per pixel)	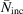 [equation (8)] (photons per pixel) (*d*_sdp_ = 0.98 cm)(*d*_g_ = 0.58 cm)	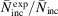 (*d*_sdp_ = 0.98 cm) (*d*_g_ = 0.58 cm)	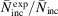 (*d*_sdp_ = 4.5 cm) (*d*_g_ = 4.1 cm)
P	9.893	3.64	2.9 × 10^6^	132.5	1.44
S	1.853	5.08	1.1 × 10^7^	35.0	0.79
Ca	0.073	3.54	3.5 × 10^7^	10.9	0.70
Fe	0.025	4.11	2.9 × 10^7^	13.2	1.18
Ni	0.010	2.83	3.4 × 10^7^	11.5	1.05

## Data Availability

Experimental data used in this manuscript can be found in our previous work (Roter *et al.*, 2026 ▸). The code we developed for our calculations can be found on Github: https://github.com/bwr0835/xray_fluor_contrast.
