# Supplementary material for: X-ray fluorescence microscopy exposure estimates using a single excitation energy
Source: J Synchrotron Radiat. 2026 Jun 16;33(Pt 4):1074–84. doi: 10.1107/S1600577526004923 (PMC13344639; doi:10.1107/S1600577526004923)
Supplement: Supplementary file 1 [file s-33-01074-sup1.pdf]

JOURNAL OF SYNCHROTRON RADIATION

Volume 33 (2026)

Supporting information for article:

X-ray fluorescence microscopy exposure estimates using a single excitation energy

Benjamin Roter, Andrew M. Crawford, Thomas V. O'Halloran and Chris Jacobsen

Supporting information for the article:

X-ray fluorescence microscopy exposure estimates using a single excitation energy

Benjamin Roter[a], Andrew M. Crawford[b,c], Thomas V. O'Halloran[b,c,d,†], and Chris Jacobsen[e,a,f,†]

[†]Corresponding authors

[a]Applied Physics Program, Northwestern University, Evanston, IL 60208, USA, [b]Department of Microbiology, Genetics & Immunology, Michigan State University, East Lansing, MI 48824, USA, [c]Department of Chemistry, Michigan State University, East Lansing, MI 48824, USA, [d]Elemental Health Institute, Michigan State University, East Lansing, MI 48824, USA, [e]Department of Physics and Astronomy, Northwestern University, Evanston, IL 60208, USA, [f]Chemistry of Life Processes Institute, Northwestern University, Evanston, IL 60208, USA

S1. Clarification on x-ray nomenclature

For simplicity in mathematical notation, in the main text, we refer to fluorescence lines by the indices ij, where i and j are electron vacancy states involved in x-ray fluorescence transitions. When photoelectric absorption leads to an initial vacancy in the $i = 1s$ state and an electron drops down so that the $j = 2p_{3/2}$ state represents the subsequent vacancy, the energy difference is released via an x-ray fluorescence photon. This fluorescence event can be described using either the IUPAC notation (Jenkins *et al.*, 1991) of K-L_3 or the equivalent Siegbahn notation (Siegbahn, 1925) of $K\alpha_1$. Additionally, when we refer to K or L_1 shell emission, K or L_1 fluorescence, or fluorescence emitted from K or L_1, we are describing x-ray fluorescence events due to vacancies being filled in the K or L_1 shell, respectively. This is less specific than referring to a particular fluorescence line like $K\alpha_1$.

S2. Total mass absorption coefficient for mixtures

In Section 3.2 of the main manuscript document, we defined the radiation skin dose D_{skin} in equations (10) and (11), which (when ignoring scattering) is proportional to a sample matrix's total mass photoionization cross section τ'_{mat}. Another approach for calculating skin dose involves the density ρ_{mat} and linear absorption coefficient μ_{mat} of the matrix material, leading to an expression of (Kirz *et al.*, 1978; Jacobsen, 2020)

$$D_{\text{skin}} = \frac{\bar{N}_{\text{inc}} E_{\text{inc}} \mu_{\text{mat}}}{\rho_{\text{mat}} A_{\text{beam}}} = \frac{E_{\text{inc}} \mu_{\text{mat}} \mathfrak{F}_{\text{inc}}}{\rho_{\text{mat}}}. \tag{S1}$$

However, the linear absorption coefficient in the absence of scattering can be written as

$$\mu_{\text{mat}} = \frac{N_A \rho_{\text{mat}}}{A_{\text{r,mat}}} \tau_{\text{mat}}, \tag{S2}$$

where

$$\tau_{\text{mat}} = 2 r_e \frac{hc}{E_{\text{inc}}} f_{2,\text{mat}}. \tag{S3}$$

In the above two equations, τ_{mat} and $A_{\mathrm{r,mat}}$ are the total atomic photoionization cross section and relative atomic mass of the matrix, respectively, r_e is the classical electron radius, N_A is Avogadro's number, h is Planck's constant, c is the speed of light in vacuum, and $f_{2,\mathrm{mat}}$ is the total imaginary part of the complex number of oscillator modes of electrons in the atoms of the matrix (Henke *et al.*, 1993; Jacobsen, 2020). Because μ_{mat} is dependent on ρ_{mat}, the matrix material's density does not actually factor into the calculated dose. Mass attenuation coefficients and cross sections are readily available in tabulations and are independent of ρ_{mat}.

In the more general case (where scattering may be present), the linear absorption coefficients $\mu_{Z'}$ for each element in the matrix can be written in terms of their corresponding mass attenuation coefficients $\mu'_{Z'}$ via

$$\mu_{Z'} = \mu'_{Z'} \rho_{Z'}, \tag{S4}$$

where

$$\mu'_{Z'} = \left(\frac{\mu}{\rho}\right)_{Z'} = \frac{N_A}{A_{\mathrm{r},Z'}} \sigma_{Z'}. \tag{S5}$$

In equation (S5), $A_{\mathrm{r},Z'}$ and $\sigma_{Z'}$ are the relative atomic mass (molar mass) and total atomic cross section for atomic number Z', respectively. [Notationally, $(\mu/\rho)_{Z'} \equiv \mu_{Z'}/\rho_{Z'}$]. The linear absorption coefficient μ_{mat} more generally also follows this form. Uniform sample matrices allow for the calculation of $\rho_{Z'}$ as (Jacobsen, 2020)

$$\rho_{Z'} = \frac{s_{Z'} A_{\mathrm{r},Z'}}{A_{\mathrm{r,mat}}} \rho_{\mathrm{mat}} = w_{Z'} \rho_{\mathrm{mat}}, \tag{S6}$$

where

$$w_{Z'} = \frac{s_{Z'} A_{\mathrm{r},Z'}}{A_{\mathrm{r,mat}}}. \tag{S7}$$

Here, $s_{Z'}$ and $w_{Z'}$ are the stoichiometric and mass fractional weighting coefficients of atomic number Z', respectively. One can obtain μ_{mat} by inserting equation (S6) into equation (S4), and summing over all $Z' \in \mathrm{mat}$. All of this yields

$$\mu_{\mathrm{mat}} = \sum_{Z' \in \mathrm{mat}} \mu_{Z'} = \rho_{\mathrm{mat}} \sum_{Z' \in \mathrm{mat}} w_{Z'} \mu'_{Z'}. \tag{S8}$$

Dividing the above equation by ρ_{mat} gives

$$\mu'_{\mathrm{mat}} \equiv \frac{\mu_{\mathrm{mat}}}{\rho_{\mathrm{mat}}} = \sum_{Z' \in \mathrm{mat}} w_{Z'} \mu'_{Z'}. \tag{S9}$$

When neglecting scattering, $\sigma_{Z'} = \tau_{Z'}$, and $\mu'_{Z'} = \left(N_A/A_{\mathrm{r},Z'}\right) \tau_{Z'} = \tau'_{Z'}$, with $\tau_{Z'}$ being the total atomic photoionization cross section for atomic number Z'. Therefore,

$$\tau'_{\mathrm{mat}} = \sum_{Z' \in \mathrm{mat}} w_{Z'} \tau'_{Z'}. \tag{S10}$$

This complements the mixture rule (Deslattes, 1969; McCullough, 1975; Jacobsen, 2020) applied to linear absorption coefficients μ_{mat}.

S3. Detector window and air effects on minimum number of photons per pixel

In Section 3.3 of the main manuscript document, we performed calculations for determining how many incident photons per pixel $\bar{N}_{\text{inc}}$ are required to detect $\bar{N}_{\text{fluor}}$ x-ray fluorescence photons at a limit of detection (LOD) of $\rho'_{\text{min}} = 0.05\ \mu\text{g/cm}^2$. We also calculated the associated minimum skin dose D_{skin} that is then delivered to the incident beam-facing matrix surface. Those calculations involved the forward model outlined Section 2.2 and accounted for the excitation dependence of photoionization partial cross sections (PCSes), Coster-Kronig (CK) transitions, and cascade effects. All of those computations were over ranges of both trace element atomic number Z and single incident photon energy E_{inc}. While we displayed the results for a windowless detector and a vacuum environment in Fig. 1, we show here in Fig. S1 the outcome of exact same calculations for $\bar{N}_{\text{inc}}$, but when accounting for a 25 µm Be window and a $d_{\text{g}} = 5$ cm air gap.

S4. Air gap estimate for experimental validation

In Section 3.5 of the main manuscript document, we noted that using a sample-to-detector plane distance of $d_{\text{sdp}} = 4.5$ cm [and therefore an air gap of $d_{\text{g}} = 4.1$ cm, following equation (21) in the main document] gave better agreement between our calculations and experiment, leading to an experimental validation ratio $\bar{N}_{\text{inc}}^{\text{exp}}/\bar{N}_{\text{inc}}$ nearer to 1 for all elements. Our previous estimate of $d_{\text{sdp}} = 0.98$ cm was not directly measured because of interference from a collimator; instead, it was inferred from an inverse-square law fit performed during our earlier experiment [see both the main text and Section S3 of Supporting Information in (Roter *et al.*, 2026)]. The distance d_{sdp} affects both the detector solid angle of acceptance Ω and attenuation of fluorescence signals over the in-air distance d_{g}. Larger values of d_{g} lead to stronger attenuation of lower energy fluorescence lines from lower-Z elements. We show in Fig. S2 the ratio $\bar{N}_{\text{inc}}^{\text{exp}}/\bar{N}_{\text{inc}}$ of experimental to calculated results for incident flux for a range of values of d_{sdp}. This figure shows that we obtain better agreement between experiment and theory, including less of a strong dependence on fluorescence photon energy, with an estimate of $d_{\text{sdp}} = 4.5$ cm instead of 0.98 cm. We therefore show the ratio $\bar{N}_{\text{inc}}^{\text{exp}}/\bar{N}_{\text{inc}}$ for $d_{\text{sdp}} = 4.5$ cm in Table 2.

References

Deslattes, R. D. (1969). *Acta Crystallographica A*, **25**, 89–93.
Henke, B. L., Gullikson, E. M. & Davis, J. C. (1993). *Atomic Data and Nuclear Data Tables*, **54**, 181–342.
Jacobsen, C. (2020). *X-ray Microscopy*. Cambridge, UK: Cambridge University Press.
Jenkins, R., Manne, R., Robin, R. & Senemaud, C. (1991). *Pure and Applied Chemistry*, **63**(5), 735–746.
Kirz, J., Sayre, D. & Dilger, J. (1978). *Annals of the New York Academy of Sciences*, **306**, 291–305.
McCullough, E. C. (1975). *Medical Physics*, **2**(6), 307–320.
Roter, B., Crawford, A. M., Jin, Q., Glowacki, A. T., Lai, B., Marin, F. S., Maxey, E., Shi, X., Culotta, V. C., Wildeman, A. S., Patel, N. K., O'Halloran, T. V. & Jacobsen, C. (2026). *Journal of Synchrotron Radiation*, **33**, 319–330.
Schoonjans, T., Brunetti, A., Golosio, B., Sánchez Del Rio, M., Solé, V. A., Ferrero, C. & Vincze, L. (2011). *Spectrochimica Acta B*, **66**, 776–784.
Siegbahn, M. (1925). *The Spectroscopy of X-rays*. London, UK: Oxford University Press.

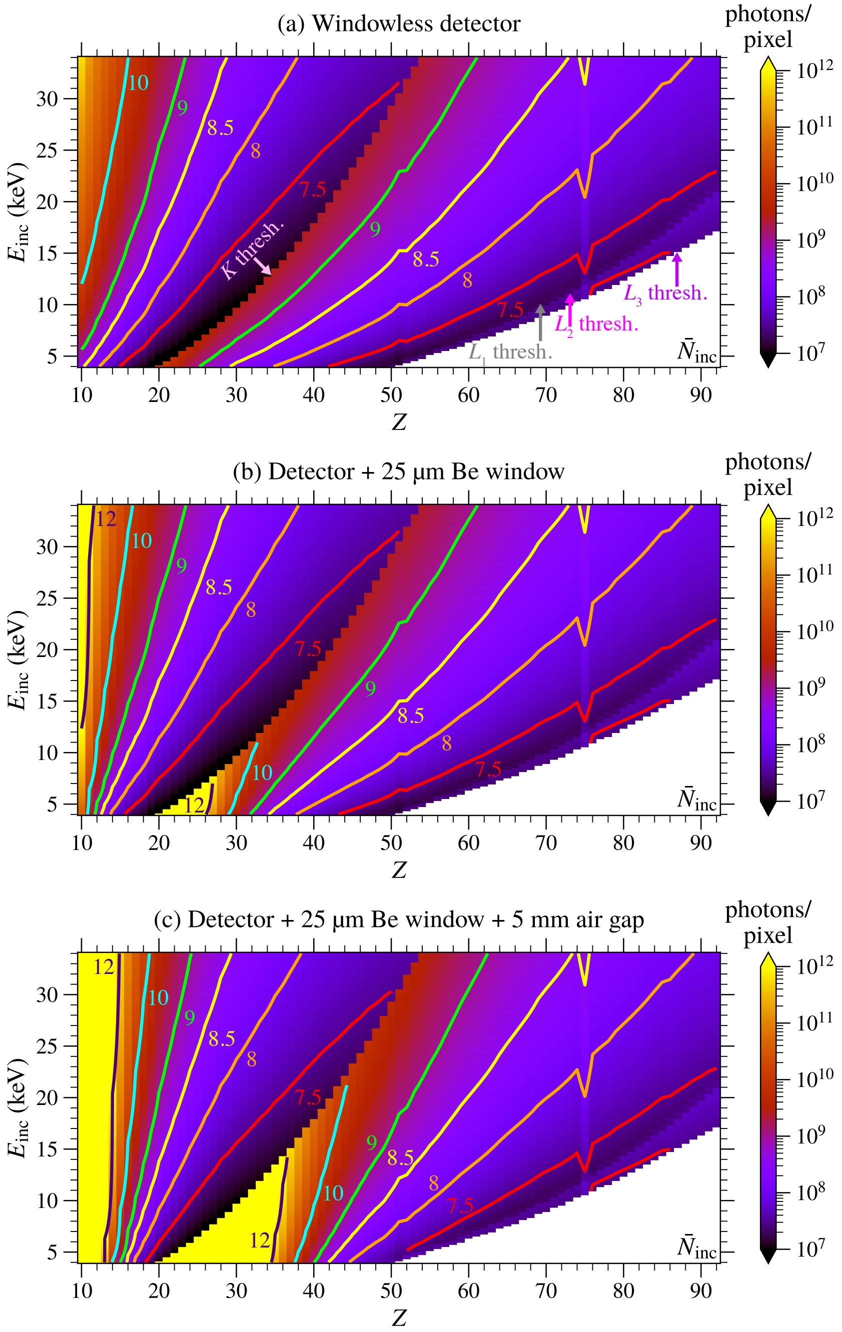

Note: Contour values correspond to base-10 exponents.

Figure S1: Effects of EDS detector windows and air on the minimum expected number of incident photons $\bar{N}_{\mathrm{inc}}$ per pixel required to detect $\bar{N}_{\mathrm{fluor}} = 5$ x-ray fluorescence photons per pixel. At top (*a*) is the combined false color map and contour plot of Fig. 1(*a*) when neglecting fluorescence signal absorption both in air and in a window mounted onto the front of an energy-dispersive (EDS) detector. At center (*b*) is the same type of false color map-contour plot combination when accounting for only a 25 µm Be window. The bottom map-contour plot combination (*c*) shows the effects of both the Be window and a $d_{\mathrm{g}} = 5$ cm air gap between the sample and the detector window. The addition of the window lead to higher $\bar{N}_{\mathrm{inc}}$ for lower-Z elements. The inclusion of the air gap further increased $\bar{N}_{\mathrm{inc}}$ for an even wider range of low-Z elements. Tabulated data from *xraylib* (Schoonjans *et al.*, 2011).

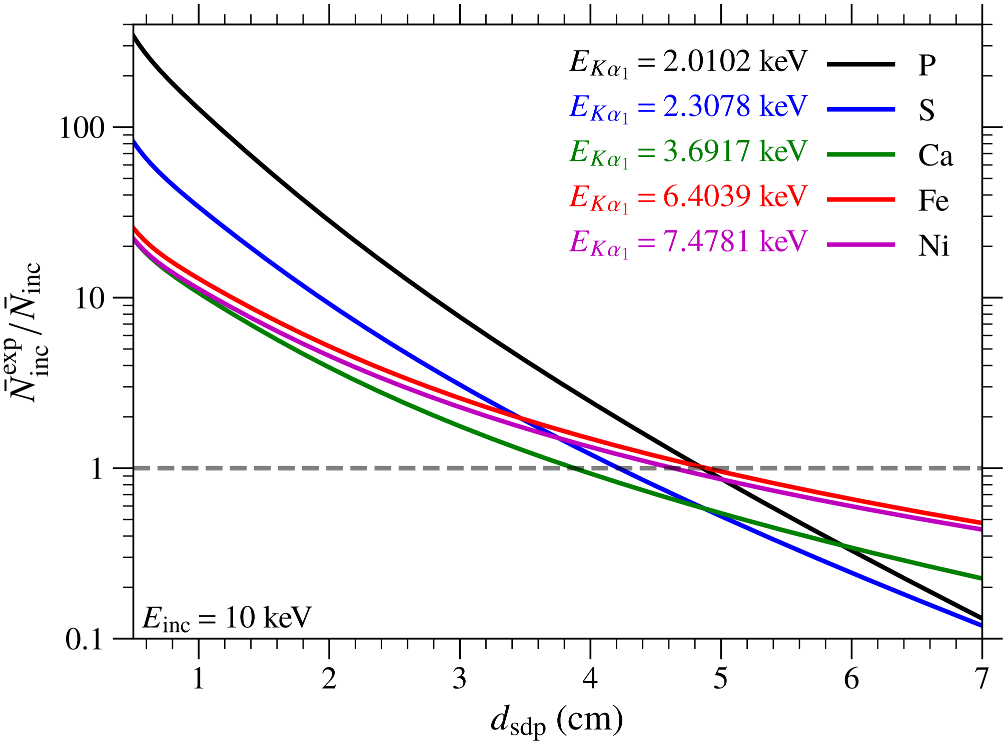

Figure S2: The experimental verification ratio $\bar{N}_{\mathrm{inc}}^{\mathrm{exp}} / \bar{N}_{\mathrm{inc}}$ versus sample-to-detector plane distance d_{sdp}. This distance was, unfortunately, not directly measured; our earlier estimate was $d_{\mathrm{sdp}} = 0.98$ cm using the inverse square law while varying d_{sdp}. We show here the ratio $\bar{N}_{\mathrm{inc}}^{\mathrm{exp}} / \bar{N}_{\mathrm{inc}}$ for each element we investigated from our previous experiment (Roter *et al.*, 2026) when accounting for x-ray fluorescence absorption in air. Lower-Z elements (thus, lower-energy XRF photons) both exhibited higher discrepancies at $d_{\mathrm{sdp}} = 0.5$ cm ($d_{\mathrm{g}} = 0.1$ cm) and dropped more quickly with d_{sdp} relative to higher-Z elements. The $K\alpha_1$ lines for each element are also shown to highlight these differences. The region between $d_{\mathrm{sdp}} = 4$ cm and $d_{\mathrm{sdp}} = 5$ cm displayed the most consistency in the discrepancies; thus, we chose $d_{\mathrm{sdp}} = 4.5$ cm as an improved estimate for the sample-to-detector distance d_{sdp} in Table 2 of the main text.
